# Supplementary material for: A data-independent acquisition-based global phosphoproteomics system enables deep profiling
Source: Nat Commun. 2021 May 5;12:2539. doi: 10.1038/s41467-021-22759-z (PMC8099862; doi:10.1038/s41467-021-22759-z)
Supplement: Supplementary file 1 — Supplementary Information [file 41467_2021_22759_MOESM1_ESM.pdf]

Supplementary Information

A Data-independent Acquisition-based Global Phosphoproteomics System Enables Deep Profiling

Reta Birhanu Kitata[1], Wai-Kok Choong[2], Chia-Feng Tsai[3], Pei-Yi Lin[1], Bo-Shiun Chen[4], Yun-Chien Chang[4], Alexey I. Nesvizhskii[5], Ting-Yi Sung[2], Yu-Ju Chen[1, 4*]

Supplementary Figure 1

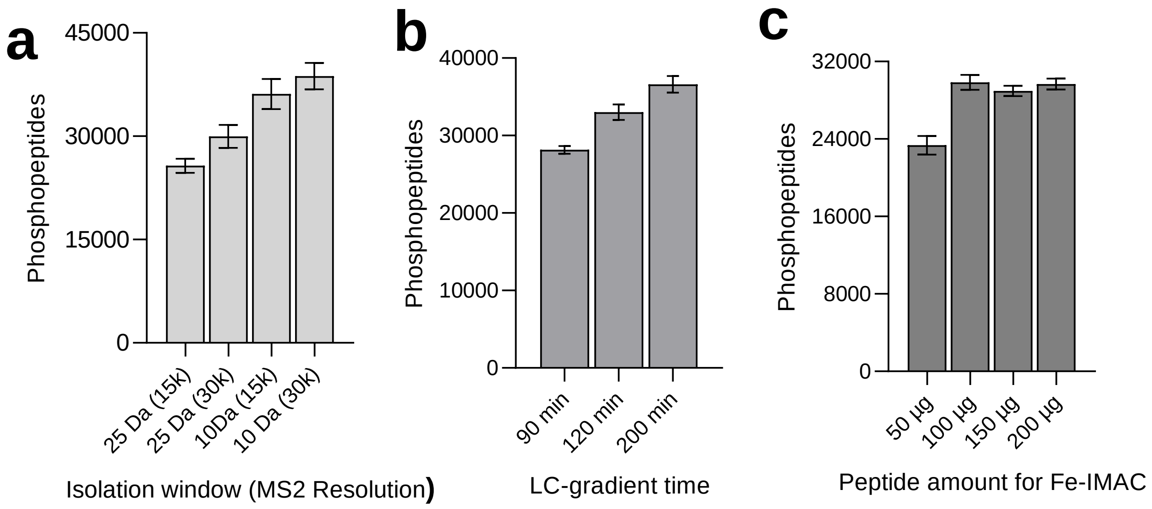

Supplementary Figure 1. **Evaluation of DIA data acquisition parameters**. The phosphopeptides enriched from NSCLC PC9 cell lysate peptide were used to evaluate different parameters. Each sample was analyzed in triplicate and library-based DIA was performed by Spectronaut against the spectral library and shown as mean ± SD from n=3 measurements. (**a**) Comparison of two different isolation windows settings (10 Da and 25 Da) and MS/MS resolution settings, 15,000 (shown as 15k) and 30,000 (shown as 30k). (**b**) Comparison of LC-gradient time. (**c**) Comparison of loading peptide amount for immobilized metal affinity chromatography (IMAC) enrichment. A narrow window of 10 Da and 30,000 MS/MS resolution resulted in best identification. gradient time of 120 min and 200 µg peptide were selected for further data acquisition. Source data are provided as a Source Data file.

Supplementary Figure 2

a

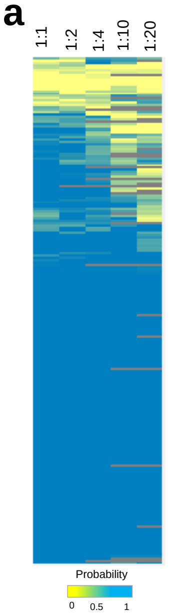

b

Gene	diphosphorylated	1:1		1:2		1:4		1:10		1:20	
EML4	AS(ph)PS(ph)PQPSSQPLQIHR(+2)	1	1	1	1	1	1	1	1	1	1
EGFR	ELVEPLT(ph)PS(ph)GEAPNQALLR(+3)	1	1	1	1	1	1	1	1	1	1
EGFR	GSHQIS(ph)LDNPDY(ph)QQDFFPK(+3)	1	1	1	1	1	1	1	1	1	1
RB1	IPGGNIYIS(ph)PLKS(ph)PYK(+2)	1	1	1	1	1	1	1	1	1	1
NOS3	IRT(ph)QSFS(ph)LQER(+3)	1	1	1	1	1	1	1	1	1	1
NOS3	IRTQS(ph)FS(ph)LQER(+2)	1	1	1	1	1	1	1	1	1	1
EIF5B	KQS(ph)FDDNDS(ph)EELEDK(+2)	1	1	1	1	1	1	1	1	1	1
RUNX1	RFT(ph)PPSTALS(ph)PGK(+2)	1	1	1	1	1	1	1	1	1	1
ERBB2	SPLAPS(ph)EGAGS(ph)DVFDGDLGMGAAK(+3)	1	1	0.995	1	1	1	1	1	0.998	1
PALM	TEVVMNS(ph)QQT(ph)PVGTPK(+3)	1	1	1	1	1	1	1	1	0.997	1
PALM	TEVVMNSQQT(ph)PVGT(ph)PK(+2)	1	1	1	1	1	1	1	1	1	1

c

Gene	triphosphorylated	1:1			1:2			1:4			1:10			1:20		
AHNAK	AS(ph)LGS(ph)LEGEAEAEASS(ph)PK(+2)	1	1	1	1	1	0.993	1	1	0.962	-	-	-	-		
CLDN18	AVS(ph)YHAS(ph)GHS(ph)VAYKPGGFK(+3)	1	0.589	0.411	1	1	0.999	1	1	1	1	0.765	0.883	1	0.016	0.987
CLDN18	IYDGGART(ph)EDEVQS(ph)YPS(ph)K(+3)	0.028	0.998	0.999	0.765	0.997	0.999	0.878	1	1	0.999	1	1	0.001	1	1
HMGA1	KLEKEEEEGIS(ph)QES(ph)S(ph)EEEQ(+3)	1	1	1	1	1	1	1	1	1	-	-	-	-		
SRRM2	SGS(ph)S(ph)QELDVKPSAS(ph)PQER(+3)	0.991	1	1	1	1	1	1	1	1	0.999	1	1	0.997	1	1
NDRG2	SRT(ph)AS(ph)LTSAAS(ph)VDGNR(+2)	1	1	1	0.997	1	0.994	0.997	1	0.998	0.995	1	0.305	0.998	0.999	0.262

Supplementary Figure 2. DIA performance evaluation for multiple-phosphorylated peptides. (**a**) Overall phosphosite localization across dilution series of quantified 222 precursors with average of the localization data shown (n=3 measurements). Site-specific localization of (**b**) diphosphorylated; (**c**) triphosphorylated peptides. Source data are provided as a Source Data file.

Supplementary Figure 3

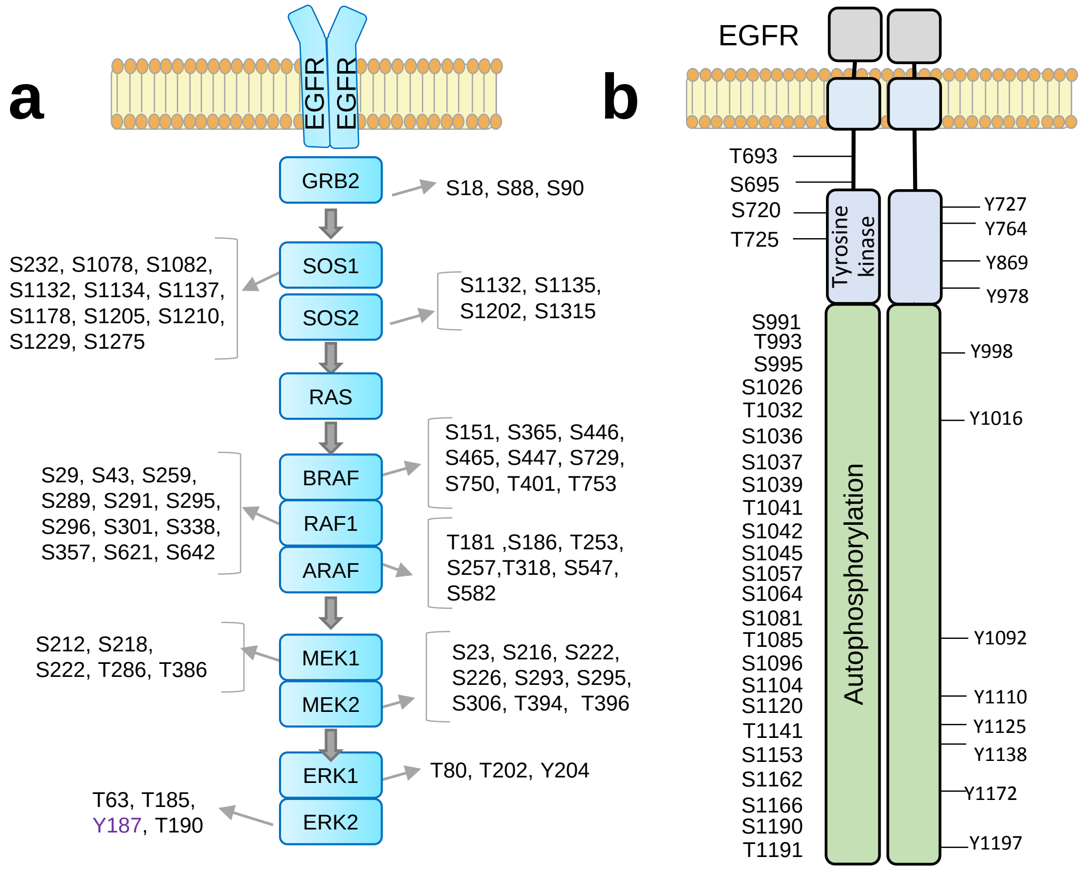

Supplementary Figure 3. RTK-RAS/RAF pathway phosphosite coverage. (**a**) Phosphosites covered in library from RTK-RAS/RAF pathway. Among total of 169 identified sites, 101 were class 1 including 14 tyrosine sites with class 1 sites shown in the figure. (**b**) Phosphosite coverage (class 1) of EGFR. Among 55 phosphosites identified, 40 were localized as class 1 including 12 tyrosine phosphorylation. Source data are provided as a Source Data file.

Supplementary Figure 4

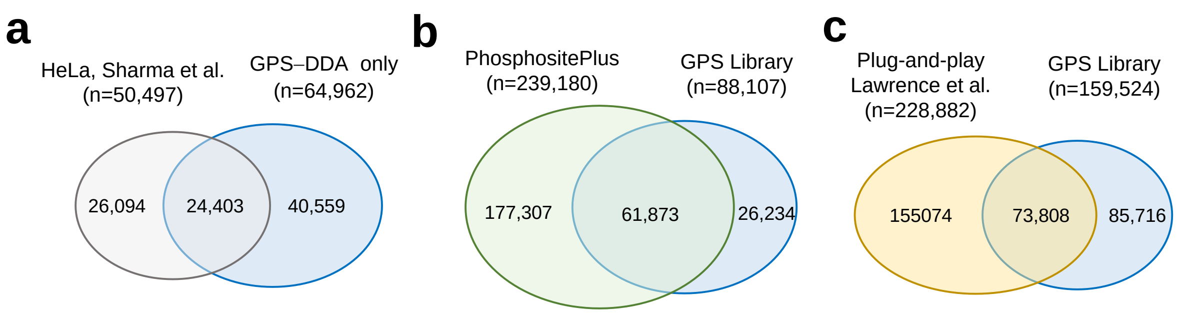

Supplementary Figure 4. Comparison of global phosphoproteome system library with large-scale phosphoproteomics reports. (**a**) Comparison of phosphosites identified from HeLa by Sharma et al. (Ref. 1) and our datasets obtained by DDA-only data from GPS. (**b**) Comparison of phosphosites from PhosphositePlus database (https://www.phosphosite.org/) accessed on 2020/10/23 and GPS library. (**c**) Comparison of phosphopeptide from GPS library with "Plug-and-play" library by Lawrence et.al. (Ref. 2). Source data are provided as a Source Data file.

Supplementary Figure 5

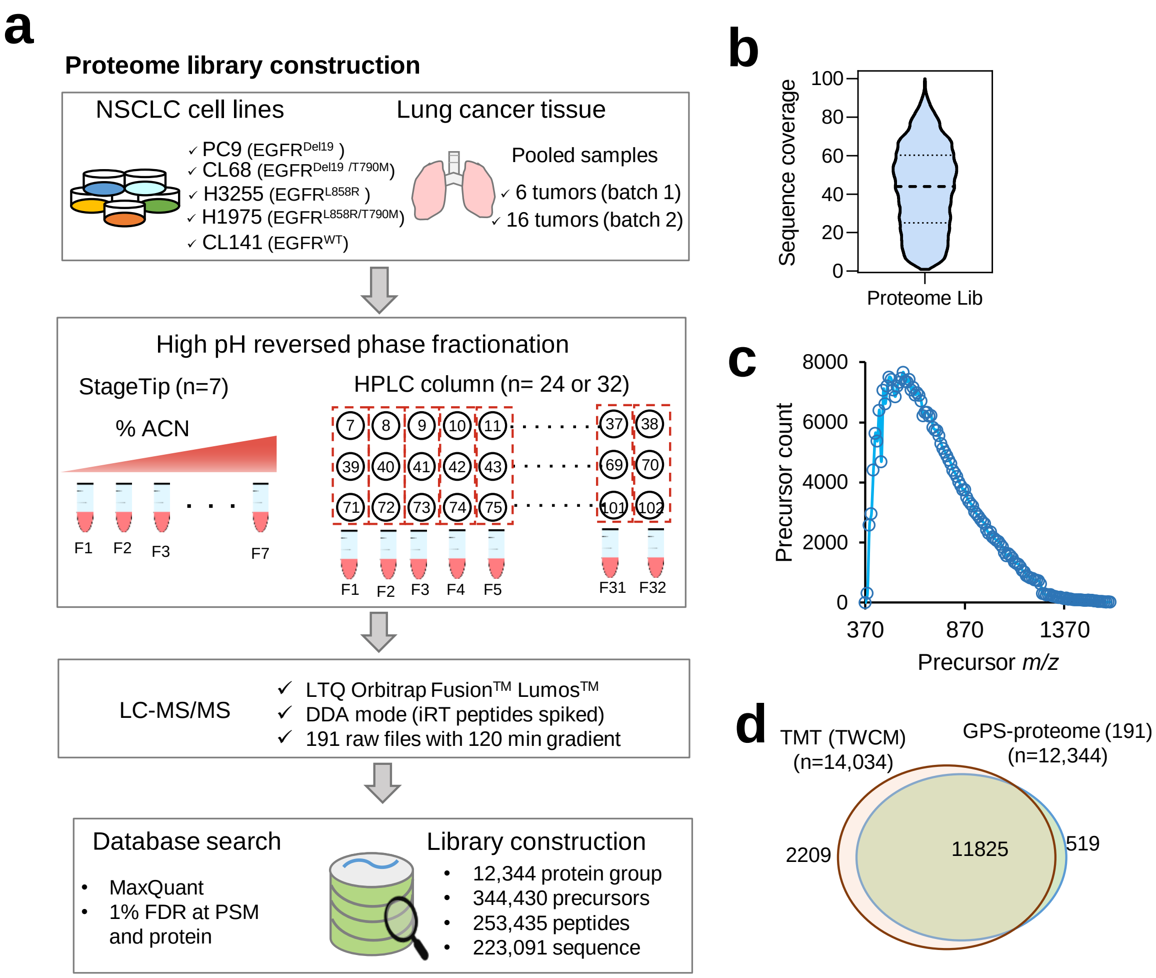

Supplementary Figure 5. Pipeline for construction of proteome spectral library. (**a**) Proteome library generation using protein extracted from five non-small cell lung cancer cell lines and twenty-two pooled lung tumor tissues. (**b**) Proteome sequence coverage distribution with a median sequence coverage of 44%. (**c**) Peptide precursor distribution (**d**) Comparison of GPS proteome library with proteome profiling of large-scale Taiwan Cancer Moonshot (TWCM) Tandem-mass tag (TMT)-based lung cancer tissue profiling by Chen et. al. (Ref. 3). Source data are provided as a Source Data file.

Supplementary Figure 6

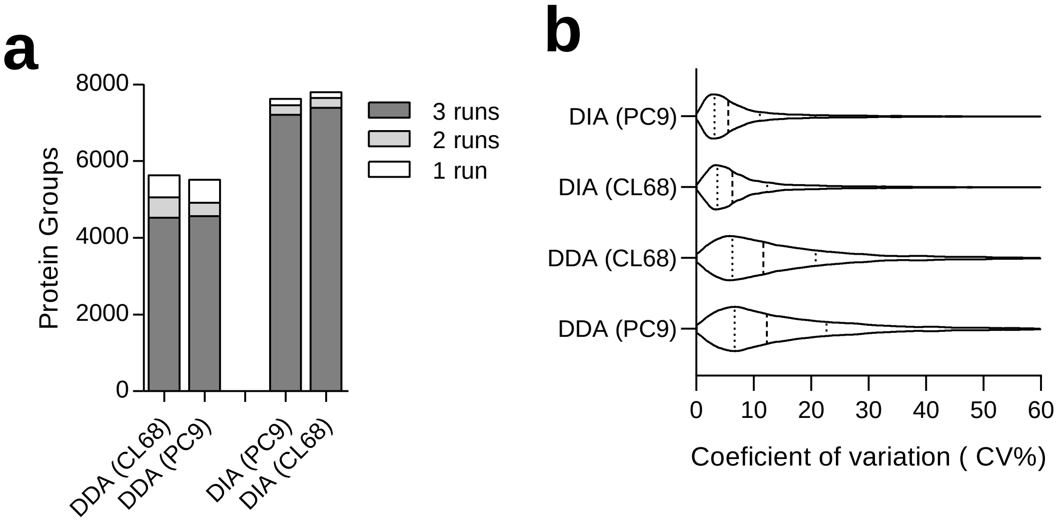

Supplementary Figure 6. Quantification performance of proteome library-based DIA and DDA in CL68 and PC9 cells. (**a**) Comparison of protein identification from PC9 and CL68 cell lines using either DDA or library-based DIA. (**b**) Comparison of coefficient of variation (CV%) with quartile shown by light dot and median values show as dark dot line. Data acquisition for PC9 and CL68 were carried out in both DDA and DIA mode (n=3 measurements). MaxQaunt LFQ search was performed for DDA (1% FDR at PSM & Protein), while targeted DIA signal extraction against the proteome library we performed by Spectronaut (1% FDR at precursor, protein levels). Overall an average protein of 4935, 5070, 7427 and 7620 were quantified in PC9 DDA, CL68 DDA, PC9 DIA and CL68 DIA, respectively. A median CV value of 6% in DIA and 12% in DDA were observed (n=3 measurements). Source data are provided as a Source Data file.

Supplementary Figure 7

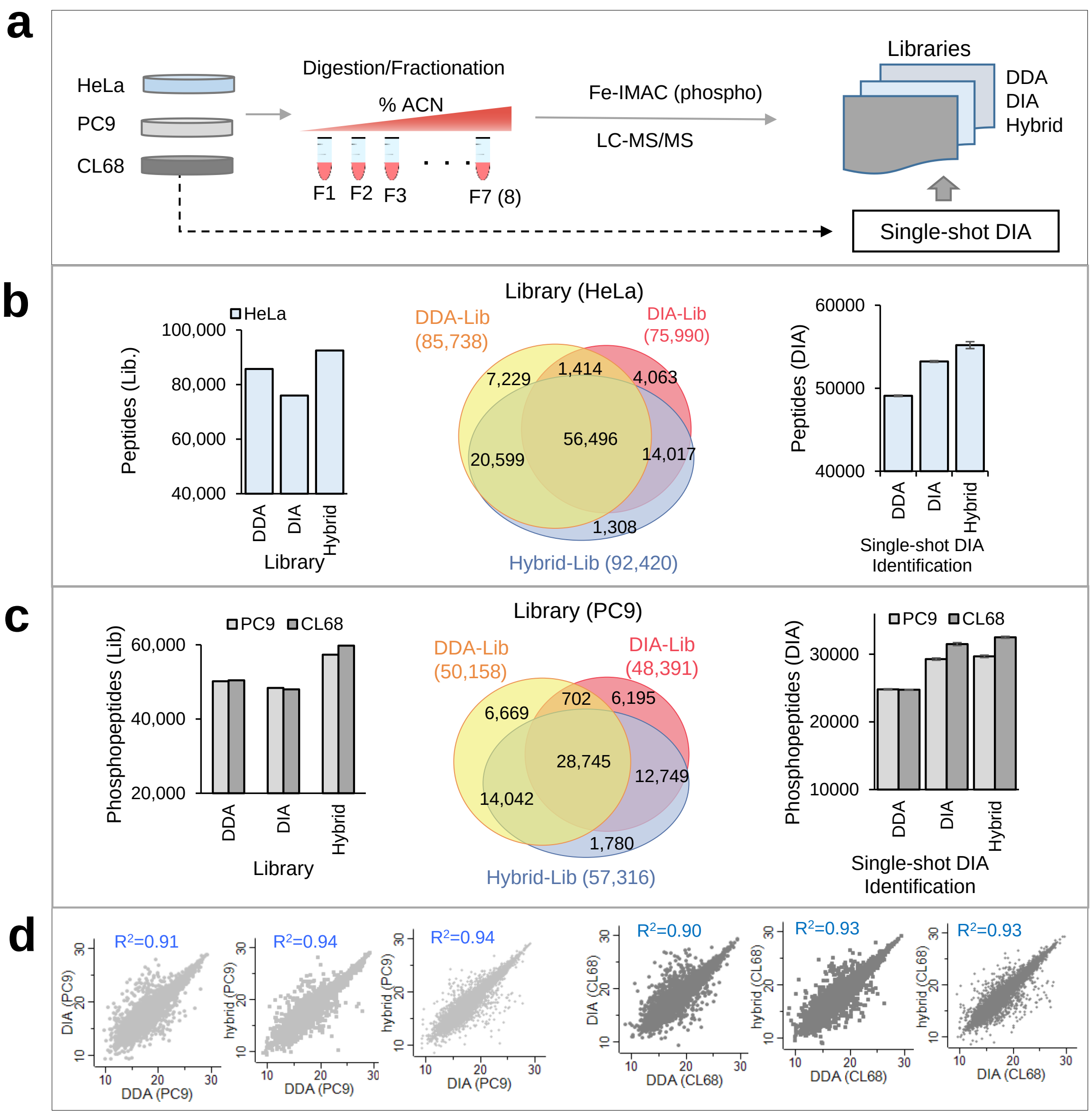

Supplementary Figure 7. Comparison on the performance of hybrid library, DDA-based and DIA-based libraries. (**a**) Experimental design to generate single mode and hybrid library from fractionated peptides followed by single-shot DIA analysis to evaluate quantification performance of the libraries. (**b**) Proteome library constructed from HeLa cells and DIA quantification against DDA, DIA and hybrid libraries. Spectral libraries were constructed from 16 raw files of fractionated HeLa peptides for each DDA, DIA and hybrid libraries. DIA quantification was done from n=3 measurements with mean ± SD shown. (**c**) Phosphopeptides library and DIA quantification against DDA, DIA and hybrid libraries for PC9 and CL68 cells. Phosphopeptides libraries were constructed from 14 raw files of fractionated and IMAC enriched PC9 and CL68 cells for each DDA, DIA and hybrid libraries. DIA quantification was done from n=3 measurements with mean ± SD shown. (**d**) Quantification correlation of phosphopeptides from the different libraries. Triplicate single-shot DIA was then acquired to evaluate quantification performance of the libraries. Source data are provided as a Source Data file.

Supplementary Figure 8

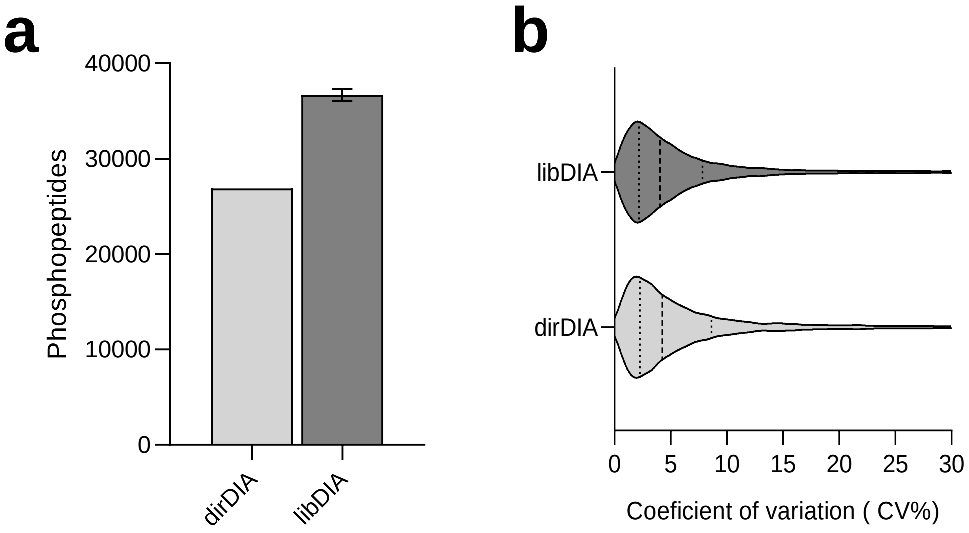

Supplementary Figure 8. Quantitative phosphoproteomics performance in breast cancer cell line using DIA. (**a**) Quantified phosphopeptides by dirDIA and libDIA from MDA-MB-453 cell line using GPS library. DIA quantification was done from n=3 measurements with mean ± SD shown. (**b**) Coefficient of variation (CV%) from triplicate runs. A median CV of 4.3% and 4.1 % for dirDIA and libDIA were achieved, respectively. Source data are provided as a Source Data file.

Supplementary Figure 9

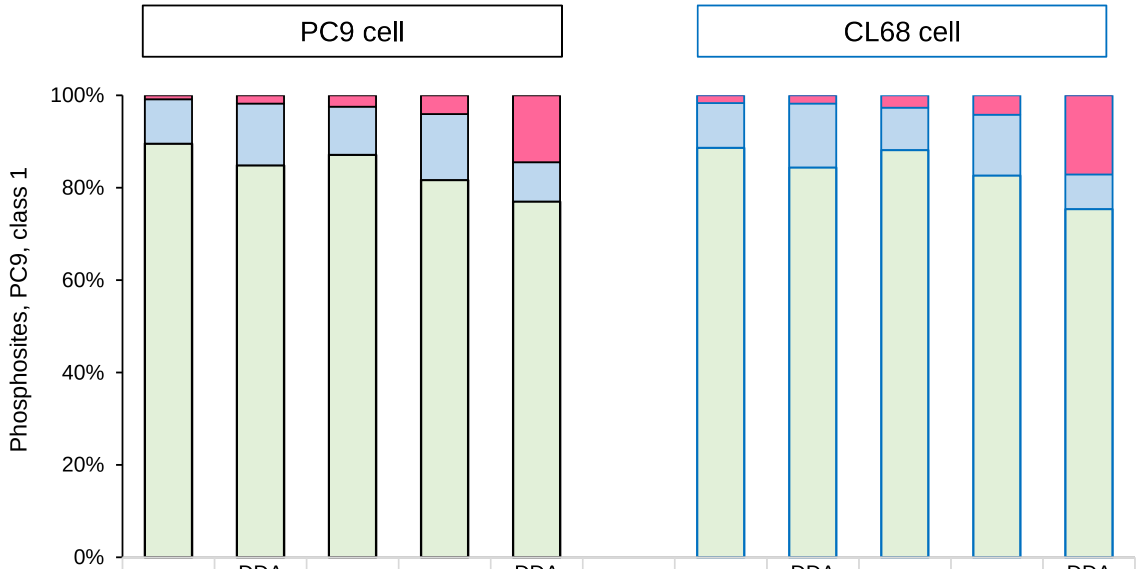

	DDA	DDA (RP)	dirDIA	libDIA	DDA (+PV)	DDA	DDA (RP)	dirDIA	libDIA	DDA (+PV)
pTyr	93	337	242	721	1721	202	456	295	834	2120
pThr	1,033	2,487	1,012	2,541	1,011	1,155	3,473	1,004	2593	920
pSer	9,642	15,807	8,492	14,548	9,145	10,616	21,233	9,672	16327	9,319

Supplementary Figure 9. Comparison of tyrosine phosphosites identification results across different acquisition modes. These class 1 phosphosites were obtained by single-shot DDA, fractionated DDA, direct DIA, library-based DIA and pervanadate (PV) treated samples annotated in the figure as DDA, DDA (RP), dirDIA, libDIA and DDA (+PV), respectively were shown. The number at the bottom of bar graph shows phosphosites of Serine (pSer), threonine (pThr), and tyrosine (pTyr). Source data are provided as a Source Data file.

Supplementary Table 1

Gene	Position	P01T/N TMT	P01T/N DIA	P02/N TMT	P02T/N DIA	P03T/N TMT	P03T/N DIA	P04T/N TMT	P04T/N DIA	P05T/N TMT	P05T/N DIA
BRAF	S446	0.90	1.30	2.20	1.45	0.82	1.26	0.71	1.48	1.25	2.53
EGFR	T693	0.68	0.15	0.56	0.42	0.89	0.51	0.32	0.22	0.95	0.41
PML	S403	1.23	0.41	1.22	1.65	1.03	2.13	1.34	1.64	0.70	0.75
CTND1	S252	0.90	0.85	1.60	1.72	0.98	0.50	0.78	0.77	0.82	0.49
ERBB3	S686	0.54	0.22	0.72	0.39	1.49	1.28	0.56	0.74	1.10	0.80
ERBB3	S982	0.41	0.11	0.80	0.31	1.15	0.85	0.54	0.47	0.84	0.39
PML	S518	0.93	1.22	1.88	2.29	0.92	0.66	1.04	1.24	0.85	0.98

Supplementary Table 1. Comparison of phosphosite ratios (tumor/normal) obtained by tandem mass tag (TMT) and DIA approaches. The TMT data was obtained from Chen et.al. (Ref. 3). Red and green color shows upregulation and down-regulation, respectively. Source data are provided as a Source Data file.

Supplementary References

1. Sharma, K. et al. Ultradeep human phosphoproteome reveals a distinct regulatory nature of Tyr and Ser/Thr-based signaling. *Cell Rep.* **8**, 1583-1594 (2014).

2. Lawrence, R.T., Searle, B.C., Llovet, A. & Villen, J. Plug-and-play analysis of the human phosphoproteome by targeted high-resolution mass spectrometry. *Nat. Methods* **13**, 431-434 (2016).

3. Chen, Y.-J. et al. Proteogenomics of Non-smoking Lung Cancer in East Asia Delineates Molecular Signatures of Pathogenesis and Progression. *Cell* **182**, 226-244.e217 (2020).
