# Supplementary material for: A data-independent acquisition-based global phosphoproteomics system enables deep profiling
Source: Nat Commun. 2021 May 5;12:2539. doi: 10.1038/s41467-021-22759-z (PMC8099862; doi:10.1038/s41467-021-22759-z)
Supplement: Supplementary file 3 — Description of Additional Supplementary Files [file 41467_2021_22759_MOESM3_ESM.docx]

**Description of Additional Supplementary Files**

**Supplementary Data 1**: Information on NSCLC Cell lines and lung cancer tissues

**Supplementary Data 2**: Information and quantification result of 166 synthetic phosphopeptides

**Supplementary Data 3**: Summary of phosphoproteome library information

**Supplementary Data 4**: Summary of quantification results from single-shot and fractionation DDA

**Supplementary Data 5**: Summary of quantification results from library-based DIA and direct DIA methods

**Supplementary Data 6**: Summary of quantitative comparison between EGFR-TKI sensitive (PC9) and resistant (CL68) cell lines

**Supplementary Data 7**: Summary of phosphoproteome profiling in lung cancer tissues

**Supplementary Data 8**: Detail of raw files uploaded to jPOST/PRIDE

**Source Data**: Raw data for underlying display items.
