# Supplementary material for: A data-independent acquisition-based global phosphoproteomics system enables deep profiling
Source: Nat Commun. 2021 May 5;12:2539. doi: 10.1038/s41467-021-22759-z (PMC8099862; doi:10.1038/s41467-021-22759-z)
Supplement: Supplementary file 12 — Reporting Summary [file 41467_2021_22759_MOESM12_ESM.pdf]

nature research

Corresponding author(s): Yu-Ju Chen

Last updated by author(s): 2021-03-23

Reporting Summary

Nature Research wishes to improve the reproducibility of the work that we publish. This form provides structure for consistency and transparency in reporting. For further information on Nature Research policies, see our Editorial Policies and the Editorial Policy Checklist.

Statistics

For all statistical analyses, confirm that the following items are present in the figure legend, table legend, main text, or Methods section.

n/a	Confirmed	
[ ]	[x]	The exact sample size (*n*) for each experimental group/condition, given as a discrete number and unit of measurement
[ ]	[x]	A statement on whether measurements were taken from distinct samples or whether the same sample was measured repeatedly
[ ]	[x]	The statistical test(s) used AND whether they are one- or two-sided<br>*Only common tests should be described solely by name; describe more complex techniques in the Methods section.*
[ ]	[x]	A description of all covariates tested
[ ]	[x]	A description of any assumptions or corrections, such as tests of normality and adjustment for multiple comparisons
[ ]	[x]	A full description of the statistical parameters including central tendency (e.g. means) or other basic estimates (e.g. regression coefficient) AND variation (e.g. standard deviation) or associated estimates of uncertainty (e.g. confidence intervals)
[x]	[ ]	For null hypothesis testing, the test statistic (e.g. *F*, *t*, *r*) with confidence intervals, effect sizes, degrees of freedom and *P* value noted<br>*Give P values as exact values whenever suitable.*
[x]	[ ]	For Bayesian analysis, information on the choice of priors and Markov chain Monte Carlo settings
[ ]	[x]	For hierarchical and complex designs, identification of the appropriate level for tests and full reporting of outcomes
[ ]	[x]	Estimates of effect sizes (e.g. Cohen's *d*, Pearson's *r*), indicating how they were calculated

Our web collection on statistics for biologists contains articles on many of the points above.

Software and code

Policy information about availability of computer code

Data collection	Mass spectrometry data were collected using LTQ Orbitrap Fusion Lumos Tribrid mass spectrometer (Thermo Fisher Scientific) coupled with ar Ultimate 3000 RSLCnano system (Thermo Fisher Scientific) and associated software.
Data analysis	MaxQuant (v1.5.3.30) software was used for database search. Spectronaut (Biognosys, v14) was used for spectral library construction and DIA data analysis. Perseus (1.6.1.1) was used for statistical tests of quantification data. The pLOGO software (v1.2.0) was used to generate motif logo. A in-house written R program used for phosphorylation site-specific quantification was uploaded to jPOST repository and can be accessed with the identification number PXD019797 in ProteomeXchange and JPST000859 in jPOST.

For manuscripts utilizing custom algorithms or software that are central to the research but not yet described in published literature, software must be made available to editors and reviewers. We strongly encourage code deposition in a community repository (e.g. GitHub). See the Nature Research guidelines for submitting code & software for further information.

Data

Policy information about availability of data

All manuscripts must include a data availability statement. This statement should provide the following information, where applicable:

- Accession codes, unique identifiers, or web links for publicly available datasets
- A list of figures that have associated raw data
- A description of any restrictions on data availability

The mass spectrometry raw datasets, reference libraries, Spectronanut quantification outputs, and MaxQuant search results were deposited in Japan ProteOme Standard Repository (jPOST; http://repository.jpostdb.org/) and can be accessed through ProteomeXchange (http://www.proteomexchange.org/). For phosphoproteome datasets, the accession numbers are PXD019797 in the ProteomeXchange and JPST000859 in the jPOST, respectively. For proteome datasets, the accession number is PXD019916 in the ProteomeXchange and JPST000864 in the jPOST, respectively. The protein sequence fasta file was obtained from UniProtKB/ [illegible]

Swiss-Prot database (https://www.uniprot.org/) and downloaded for "Homo Sapiens" (December 2015); and for "S. Cerevisiae." (February, 2018). The iRT peptides fasta file was downloaded from Biognosys website (https://biognosys.com/shop/irt-kit#SupportMaterials). For functional and family annotation the following databases were used: STRING database (version 11). (https://string-db.org/); KEGG database (https://www.genome.jp/kegg/), PhosphoSitePlus database (https://www.phosphosite.org/); human dephosphorylation database, DEPOD (www.depod.org) and kinase families of KinMap database (http://www.kinhub.org/kinmap/).

Field-specific reporting

Please select the one below that is the best fit for your research. If you are not sure, read the appropriate sections before making your selection.

☒ Life sciences ☐ Behavioural & social sciences ☐ Ecological, evolutionary & environmental sciences

For a reference copy of the document with all sections, see nature.com/documents/nr-reporting-summary-flat.pdf

Life sciences study design

All studies must disclose on these points even when the disclosure is negative.

Sample size	Five non-small cell lung cancer (NSCLC) cell lines were used to construct spectral library and two cell lines were used for DIA analysis. The cell lines were selected based on difference in EGFR mutation status and tyrosine kinase inhibitor (TKI) sensitivity and resistance. For spectra library construction, 22 tumor samples were also used to increase the proteome and phosphoproteome coverage. As a proof-of-concept, 5 pairs of tumor and adjacent normal tissues were used for DIA analysis.
Data exclusions	No data was excluded.
Replication	To demonstrate the pipeline for DIA-based phosphoproteome analysis, three technical replicates were used for every experiment in the methodology development. In the proof-of-concept lung cancer cell analysis, three biological replicates were performed for two lung cancer cell lines.
Randomization	Only two cell lines were used for the DIA analysis, randomization was not performed. For the 5 pairs of normal and adjacent samples, normal tissues are acquired first, followed by tumor tissue samples. For spectral library construction, randomization was not involved as all the datasets were merged and processed for peptide identification.
Blinding	For DIA analysis, blinding was not performed as the focus is to demonstrate the technical performance of the pipeline.

Reporting for specific materials, systems and methods

We require information from authors about some types of materials, experimental systems and methods used in many studies. Here, indicate whether each material, system or method listed is relevant to your study. If you are not sure if a list item applies to your research, read the appropriate section before selecting a response.

Materials & experimental systems

n/a	Involved in the study	
☒	☐	Antibodies
☐	☒	Eukaryotic cell lines
☒	☐	Palaeontology and archaeology
☒	☐	Animals and other organisms
☐	☒	Human research participants
☒	☐	Clinical data
☒	☐	Dual use research of concern

Methods

n/a	Involved in the study	
☒	☐	ChIP-seq
☒	☐	Flow cytometry
☒	☐	MRI-based neuroimaging

Eukaryotic cell lines

Policy information about cell lines

Cell line source(s)	The human lung adenocarcinoma cell line PC9, CL68, H3255, CL141 and H1975 were gifts from Department of Internal Medicine, National Taiwan University, Taiwan. The human cell line H1975 is available in ATCC (ATCC® CRL-5908™) and PC9 can be obtained from RIKEN BioResource Research Center (Ibaraki, Japan).The MDA-MB-231 breast cancer cell line was purchased from Bioresource Collection and Research Center, Taiwan.
Authentication	None of the cell lines were authenticated.
Mycoplasma contamination	The cell lines were tested negative for mycoplasm contamination.
Commonly misidentified lines (See ICLAC register)	No commonly misidentified cell line are used in this study

Human research participants

Policy information about studies involving human research participants

Population characteristics	The lung cancer surgical tumor tissue specimens were collected based IRB approved protocol. The 25 tissue samples have characteristics including epidermal growth factor receptor (EGFR) gene mutation (80%), stage (76% early stage, 24% late stage), and gender (64% male, 36% female). The 5 non-small cell lung cancer (NSCLC) cell lines also vary in EGFR gene mutation: PC9 (exon 19 deletion), CL68 (exon 19 deletion and T790M), H3255 (L858R), CL141 (wild type) and H1975 (L858R and T790M).
Recruitment	The cases were selected based on clinical histology including cancer staging and EGFR mutation status. The recruitment was mainly based on our previous lung cancer proteogenomics study (Cell. 2020; https://doi.org/10.1016/j.cell.2020.06.012).
Ethics oversight	All ethical regulations have been approved by Institutional Review Board (IRB) on Biomedical research of Academia Sinica and National Taiwan University Hospital Research Ethics Committee. All patients have provided written informed consent.

Note that full information on the approval of the study protocol must also be provided in the manuscript.
